# Strong SARS-CoV-2 T-Cell Responses after One or Two COVID-19 Vaccine Boosters in Allogeneic Hematopoietic Stem Cell Recipients

**DOI:** 10.3390/cells11193010

**Published:** 2022-09-27

**Authors:** Béatrice Clémenceau, Amandine Le Bourgeois, Thierry Guillaume, Marianne Coste-Burel, Pierre Peterlin, Alice Garnier, Maxime Jullien, Jocelyn Ollier, Audrey Grain, Marie C. Béné, Patrice Chevallier

**Affiliations:** 1 Nantes Université, Inserm UMR 1307, CNRS UMR 6075, Université d’Angers, CRCI2NA, F-44000 Nantes, France; 2 Hematology Department, Nantes University Hospital, F-44000 Nantes, France; 3 Virology Department, Nantes University Hospital, F-44000 Nantes, France; 4 Hematology Biology, Nantes University Hospital, F-44000 Nantes, France

**Keywords:** allogeneic hematopoietic stem cell transplantation, COVID-19, anti-SARS-CoV-2 vaccines, booster, humoral immunity, cellular immunity

## Abstract

A full exploration of immune responses is deserved after anti-SARS-CoV-2 vaccination and boosters, especially in the context of allogeneic hematopoietic stem cell transplantation (allo-HSCT). Although several reports indicate successful humoral responses in such patients, the literature is scarce on cellular specific immunity. Here, both B- (antibodies) and T-cell responses were explored after one (V3 *n* = 40) or two (V4 *n* = 12) BNT162b2 mRNA vaccine boosters in 52 allo-HSCT recipients at a median of 755 days post-transplant (<1 year *n* = 9). Results were compared with those of 12 controls who had received only one booster (BNT162b2 *n* = 6; mRNA-1273 *n* = 6). All controls developed protective antibody levels (>250 BAU/mL) and anti-spike T-cell responses. Similarly, 81% of the patients developed protective antibody levels, without difference between V3 and V4 (82.5% vs. 75%, *p* = 0.63), and 85% displayed T-cell responses. The median frequency of anti-spike T cells did not differ either between controls or the whole cohort of patients, although it was significantly lower for V3 (but not V4) patients. COVID-19 infections were solely observed in individuals having received only one booster. These results indicate that four vaccine injections help to achieve a satisfactory level of both humoral and cellular immune protection in allo-HSCT patients.

## 1. Introduction

The initial period of the COVID-19 pandemic (2019–2020) was responsible for up to 25% of deaths in recipients of allogeneic hematopoietic stem cell transplantation (allo-HSCT) [1,2]. The rapid availability of SARS-CoV-2 mRNA vaccines [3] at the end of 2020 allowed for relative protection to be provided to these patients, since up to 80% of them were shown to develop anti-spike (anti-S) antibodies after two doses of the vaccine [4,5]. For those who failed to display humoral responses, the reasons were a short delay between the graft and the vaccine (first year post-transplant), ongoing immunosuppressive treatment or profound B-cell aplasia [5,6]. Of note, in the latter studies, T-cell adaptive responses were seldom documented, although they are likely to provide the best efficacy against viral infections through cytotoxic activity against infected cells, limiting viral proliferation, as reported in other types of viral infection and suggested after SARS-CoV-2 contact [7,8]. Such studies were, however, mostly performed at a distance from allo-HSCT and the response rates reported are very heterogeneous, showing T-cell responses for 19% to 82.3% of the patients [9,10,11]. In a cohort of 46 allo-HSCT patients having received two doses of the vaccine, our group recently reported a strong response of specific anti-SARS-CoV-2- CD4+ T cells in 89% of humoral responders and 40% of nonhumoral responders [12]. These responses were associated with a predominant IFNγ/TNFα+ cytokine profile assessed by intracellular staining of stimulated T cells.

As specific humoral responses fade over time, the policy of prescribing booster vaccinations soon developed. In the population of allo-HSCT recipients, the interest of at least one booster has been confirmed. Not only do specific antibody levels increase in these anamnestic responses, but this translated to a persistent and significant reduced incidence of severe forms or deaths due to COVID-19 infection [13,14,15].

Here, humoral and T-cell responses were evaluated after one (Vaccine 3 or V3 *n* = 40) or two (V4 *n* = 12) BNT162b2 mRNA vaccine boosters in 52 allo-HSCT patients and 12 healthy donors, including 12 and 8, respectively, from our previous study after two doses of vaccines [12].

## 2. Patients and Methods

### 2.1. Patients

Blood samples were collected between 18 January and 3 March 2022 during the SARS-CoV-2 Omicron wave in France. All participants provided informed consent and the study was approved by the Ethical Review Board of Nantes University Hospital.

### 2.2. Serological Analyses

Serum antibody levels to the SARS-CoV-2 spike protein receptor-binding domain were assayed in all participants using Roche Elecsys^®^ (Rotkreuz, Switzerland). Titers ≥ 0.8 BAU/mL considered positive, the highest threshold being >250 BAU/mL. Serum dilutions were performed for positive samples in order to increase the threshold to 2500 BAU/mL.

### 2.3. Peripheral Blood Mononuclear Cell (PBMC) Analyses

PBMCs were isolated from samples collected on Ethylendiamide tetracetic acid (EDTA) by Ficoll density gradient centrifugation (Eurobio, Les Ulis, France) and frozen as reported previously [12]. Immunophenotype was determined on thawed cells by flow cytometry using Fixable Viability Stain-780 to select live cells and fluorochrome-conjugated monoclonal antibodies to CD45, CD3, CD14, CD19 and HLA-DR [12].

PBMC (2 × 10^5^) in 100 µL medium were added to each well of Human ELISpot^PRO^ Kit plates (Mabtech 3420-2AST-10, Nacka Strand, Sweden) to assess IFN-γ production. Three peptide pools, added as 100 μL to each well, covered the SARS-CoV-2 spike glycoprotein, EBV and CMVpp65, respectively. Anti-IFN-γ antibodies were added after removing the cells, and ELISpots were stained according the the manufacturer’s instructions. Spot-forming cells (SFU) were counted on a Bioreader 5000-pro-S (BIOSYS GmbH, Karben, Germany). The median background for the negative control was 0 SFU/2 × 10^5^ cells (range 0–5). Frequencies of spot-forming units (SFU) were reported per 100 CD3^+^ T cells, evaluated beforehand in each PBMC suspension.

SARS-CoV-2 T-cell Analysis Kits (PBMC) from Miltenyi Biotec (Bergisch Gladbach, Germany) were used to analyze CD4^+^ and CD8^+^ SARS-CoV-2-reactive T cells as reported before [11]. Briefly, aliquotes of 1 × 10^6^ PBMCs per well, in flat-bottom 96-well plates, were incubated with the peptide pools. Cytostim^®^ (Miltenyi Biotec) was used as a positive control of T-cell stimulation. After stimulation, brefeldin A (2 µg/mL) was added and the plates were incubated for 4 additional hours. PBMCs were then stained according to the manufacturer’s instructions for the detection of T-cell subsets (CD3, CD4, CD8 and intracytoplasmic IFN-γ and/or TNF-α and/or IL-2). A minimum of 100,000 live CD3+ T-cells were acquired on a BD LSRFortessa™ instrument (BD Biosciences). T-cell subsets were analyzed using FlowJo v.10.7.1 software (FlowJo, BD LifeSciences, Franklin Lakes, NJ, USA). Percentages obtained for the unstimulated condition were subtracted from those of stimulated conditions.

### 2.4. Statistics

Associations between clinical characteristics and immune responses were investigated using Chi², Wilcoxon, Unpaired Student t or Mann–Whitney U tests with the R software via BiostaTGV.( UMR S 1136 Inserm & Sorbonne Université, Paris, France), *p* values < 0.05 were considered statistically significant.

## 3. Results

### 3.1. Patient Characteristics

The whole cohort of patients studied included 52 allo-HSCT recipients, 40 of them having benefited from a first boost (third injection, V3). Twelve patients received a second booster (V4) because they had no humoral response (*n* = 4), a nonprotective antibody level (<250 BAU/mL) [5] (*n* = 4), or because they were more than 6 months away from the first booster (*n* = 4). Additionally, 12 controls who had received one booster (V3) agreed to provide blood samples as well.

Characteristics of the participants are summarized in Table 1.

### 3.2. Global Vaccination Characteristics

Table 2 summarizes the type of vaccines administered, vaccination schedules and delays between vaccination and analyses performed. Of note, patients who received two boosters had initiated vaccination significantly earlier after transplantation than those who only had one. This logically translated to a longer time between allo-HSCT, V3 and analyses in the latter group. Yet, the delays between V1 and boosters and V1 and analyses were not significantly different. As expected, however, the time between the last vaccine and analyses was shorter for V4 patients and for V3 controls.

### 3.3. Humoral Responses

All controls (100%) and 81% individuals of the whole patient cohort developed protective anti-S antibody levels (>250 BAU/mL) [5] after boost vaccinations. The rate of subjects reaching the highest antibody concentration (>2500 BAU/mL) was, however, significantly higher in controls than for the whole patient cohort (100% vs. 52%, *p* = 0.005) (Table 2). The median time between allo-HSCT and analyses was 755 days (range: 189–5293), with only nine patients within the first year post-transplant (Table 1), which may explain the high proportion of patients with a good humoral response.

### 3.4. Cellular Responses

**Figure 1:**
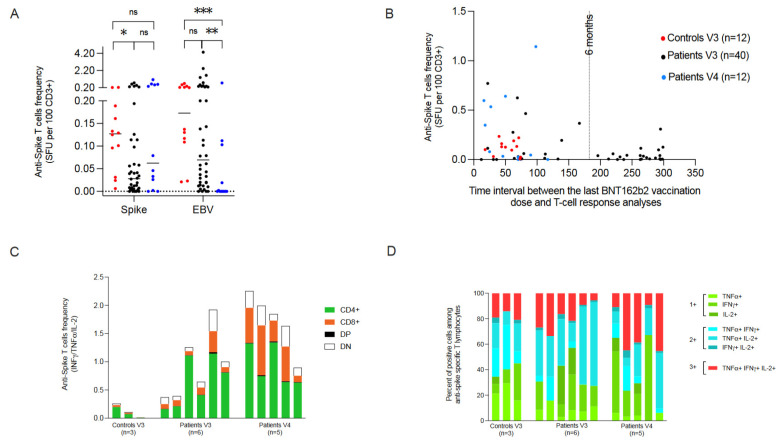
T-cell responses after 1 (V3) or 2 (V4) booster vaccination in allo-HSCT recipients and controls. (**A**) Anti-SARS-CoV-2 spike and EBV specific T-cell levels detected as IFNγ ELISpot tests (SFU per 100 CD3+ cells). Individual data and medians are shown. (**B**) Anti-SARS-CoV-2 spike T-cell frequencies (SFUs) as a function of the time interval between the last booster (V3 or V4) and analysis. The vertical bar indicates the 6-month delay between the respective vaccination and analysis. (**C**) T-cell reactivity assessed as intracytoplasmic cytokine production according to T-cell subsets (CD4+, CD8+, double-positive (DP) and double-negative (DN)) in 11 allo-HSCT recipients and 3 healthy controls with more than 0.180% SFU in ELISpot assays. (**D**) CD3+ T-cell polyfunctionality analysis. Proportions of anti-spike-specific CD3+ T cells according to the 8 intracellular cytokine profiles detected. * *p* ≤ 0.05, ** *p* ≤ 0.01, *** *p* ≤ 0.001. ns, not significant.

After booster vaccination, 100% of the controls and 85% of the patients developed specific T-cell responses. The median frequency of anti-spike T cells was lower in the patient cohort (median: 0.034 SFU/100 CD3+ T cells, range 0.000–1.143) than in controls (median: 0.127, range 0.006–0.235) although this difference was not statistically significantly different (*p* = 0.08). Yet, this median frequency was significantly lower for V3 patients compared to V3 controls (*p* = 0.03) (Table 2 and Figure 1A).

This could be explained by the immunosuppressed status of the patients but possibly also by the fact that the time interval between boost vaccination and analysis was significantly greater for patients than for controls (224 days vs. 55 days, *p* = 0.002) (Table 1). However, no significant difference in the frequency of anti-SARS-CoV-2 T cells was observed when patients were tested within or after 6 months following the last vaccination (Figure 1B). Of note, the median frequency of EBV-specific T cells, tested concomitantly as control, was 0.0695 (0.000–4.350) and 0.00 (0.000–0.750) SFU/100 CD3^+^ T cells for V3 and V4 subgroups, respectively (Figure 1A), confirming the poorer immunocompetence of the subgroup of allo-HSCT recipients who required two boosters.

Longitudinal analyses performed for five patients after V1, V2 and V3 disclosed that T-cell responses increased after V1 and V2, but decreased after V3 in four patients and was stable in one patient.

For 14 individuals with the highest frequencies of anti-spike CD3+ T cells (>0.180 SFU/100 CD3+ T cells), the Th1 cytokine profile of CD3+ T-cell subsets were assessed by INF-γ, TNF-α and IL-2 intracellular staining. In a first analysis (Figure 1C), the production of any cytokine was examined in four CD3+ T-cell subsets: CD3+/CD4+, CD3+/CD8+, CD3+/CD4+/CD8+ (double-positive, DP) and CD3+/CD4-/CD8- (double negative, DN) subsets, respectively. As already reported after two vaccinations, [11] a predominance of anti-spike CD4+ T-cell responses was observed. However, more CD8+ responses were seen in V4 patients.

Finally, cytokine secretion profiles were examined in search of polyfunctional cells (Figure 1D, Supplemental Figure S1). Seven different profiles were observed, of a single cytokine, two cytokines and three cytokines, respectively. Interestingly, a polyfunctional cytokine profile was observed in both controls (*n* = 3), and patients (V3 *n* = 6; V4 *n* = 5).

### 3.5. Follow-Up of Humoral and Cellular Specific Responses

Humoral and cellular response analysis after V2 and after booster vaccinations could be compared in 12 patients at a median time of 44 days after V2 and 248 days after V3/V4, respectively. This disclosed that: (i) 6 patients retained and 5 achieved protective antibody levels after booster(s), while 1 displayed seroconversion; and (ii) the frequency of specific Tvcells decreased for 10 of them. An increase in specific T-cell frequency occurred only for 2 patients between the two doses. Similar comparisons were also performed in eight controls (at median times of 58 days after V2 and 55 days after V3). This showed that: (i) all controls retained protective antibody levels after boosters; and (ii) the frequency of specific T cells remained stable or slightly decreased in five of them, while two had a strongly increased specific T-cell frequency after the third dose.

### 3.6. Specific Responses and Protection

Of note, there was no correlation between high or weak antibody levels and T-cell responses (75% vs. 82%, *p* = 1). No factor wad identified that would predict T-cell responses in univariate analysis (Supplemental Table S1).

Importantly, considering the 10 patients with no antibody response (*n* = 5) or not reaching a protective antibody level after boosts (*n* = 5), 8 of them (80%) displayed measurable T-cell responses. (Supplemental file: Table S2).

At the time of the last follow-up (1st June 2022), two controls with good humoral (>250 BAU/mL) and T-cell responses (0.125 and 0.235 SFU/100 CD3+ T cells) suffered from a non-severe COVID-19 infection. Similarly, two V3 patients (one with negative serology and 0.039 SFU/100 CD3+ T cells and one with >2500 BAU/mL and 0.040 SFU/100 CD3+ T cells) presented with a non-severe COVID-19 infection. One V3 patient with negative serology and 0.367 SFU/100 CD3+ T cells at the time of analysis died from a COVID-19 infection. Of note, no graft-versus-host disease (GVHD) (re)occurrence was noted in this cohort after V3 or V4.

## 4. Discussion

This study reports on the impact of one or two anti-SARS-CoV-2 booster vaccinations in allo-HSCT recipients. Although gathering a limited number of cases, this provides new information about the interest of a second booster in this setting.

This cohort shows that after one or two booster vaccinations, 81% of allo-HSCT recipients achieve a protective humoral response. This in line with other publications having reported a good rate of immunization after anti-SARS-CoV-2 vaccination in this potentially immunocompromised population [5,13]. No difference was noted between V3 and V4 patients, probably as a consequence of the high incidence of humoral responses already observed after V3 in this particular group of patients.

Of note, 85% of these patients were shown to have also developed a specific T-cell response evidenced by the detection of IFN-γ-secreting cells upon stimulation with SARS-CoV-2 spike peptides. Again, this is in line with the results provided by Kimura et al. after one booster, where a positive T-cell response was documented in around 80% of 20 allo-HSCT patients [16]. Similarly, the proportion of patients with positive T cells did not vary much between the third and fourth doses. However, T-cell frequencies were comparable between V4 patients and controls while they were significantly lower in V3 cases, suggesting the beneficial role of a second booster. Moreover, although a lower frequency of specific T cells was observed in patients when tested more than 6 months postbooster, their detection confirms their persistence as described in healthy cohorts. Indeed, it was previously shown that T-cell responses are maintained for at least 6 months following COVID-19 infection [17,18]. Here, in a subset of good responders, this response was confirmed to concern mostly CD4+ T cells, as observed in our previous report after a second vaccination [12]. Specific CD8+ cells could be evidenced in patients having received a boost more recently. This is consistent with the activation of cytotoxic effector cells with a shorter persistence and recall of CD4+ memory cells in subjects at a longer distance from the last challenge. Moreover, in all patients and controls, a subset of T cells was demonstrated to be capable of polyfunctionality, concomitantly producing two or three of the cytokines tested: IFN-γ, TNF-α and IL-2, respectively. This again is in line with the report of Kimura et al. [16], mentioned above, where a positive CD4+ T-cell response was observed in 55–80% of allo-HSCT recipients (varying based on functionality) while frequencies of CD4+ polyfunctional T cells significantly increased after the third dose. Relationships between the levels of T-cell subset responses (CD4+ and CD8+), viral clearance kinetics and asymptomatic or paucisymptomatic control of SARS-CoV-2 infection are not well-defined and the reasons are multifaceted. Indeed, such analyses are usually, as here, confined to the circulatory compartment. Evidence for protective T-cell responses would require explorations in the airways, especially in the nasal cavity, which is both the point of entry and site of initial rapid replication of the virus. Moreover, a clear hierarchy is still ill-defined, of which SARS-CoV-2 epitopes generate CD8+ and CD4+ T cells triggered with the best efficacy [19]. Additionally, SARS-CoV-2 T-cell cross-reactivity, namely the presence of memory T cells induced by previously encountered other viruses (likely related to coronavirus, but potentially any other pathogen), which may be highly variable between patients, complicates the analysis of the simple relationship between vaccine response and protection. Another important aspect that can contribute the role of T-cell responses in protection against COVID-19 infection is not related to the quantity, function or localization of T cells, but rather on the ability of SARS-CoV-2 to escape them, a point not addressed here. This evaluation might be facilitated by recently developed new methods that can provide a rapid estimation of the quantity, diversity and function of SARS-CoV-2-specific T cells [20,21].

Another interesting observation is that blood samples in this cohort were collected during the Omicron BA.1 wave in France. The challenge that constitutes variant evolution is of crucial importance regarding humoral and T-cell responses, especially in immunocompromised hosts. Recently, it was shown in healthy vaccinated populations that T-cell responses are remarkably preserved and able to cross-recognize SARS-CoV-2 variants, from Alpha to Omicron, conversely to what has been observed for SARS-CoV-2-specific antibody and B-cell responses [22,23,24]. Considering the very low incidence of severe COVID-19 infections observed in our study, it seems that this is also the case in allo-HSCT recipients, at least when they are at distance of the transplantation. Nevertheless, prudence is required as there are already data showing that Omicron BA.1 mutations in SARS-CoV-2 spike could reduce T-cell responses in vaccinated and convalescent individuals [25]. For this matter, the role of heterologous primo and boost immunization with various vaccine types should be considered, as improved immunogenicity—both humoral and cellular—has been documented compared with the use of the same vaccines all along the vaccination schedule [26,27].

Finally, no GVHD reactivation has been observed after V3 or V4 vaccines in our cohort, better even than the results shown by Kimura et al. [16] where the incidence of new onset of acute GVHD was only 5.4% and worsening of pre-existing acute GVHD was 1.4% after three doses of the vaccine.

In conclusion, strong SARS-CoV-2 T-cell responses were documented here after one or two COVID-19 vaccine boosters in allo-HSCT recipients. T-cell frequencies were comparable between V4 patients and controls while COVID-19 infections were only observed in individuals with one booster. Although these results deserve to be confirmed on a larger cohort, they indicate also that two booster vaccines may help to achieve sufficient protection in allo-HSCT recipients through persistent specific T-cell responses.

## Figures and Tables

**Table 1:** Patient and control characteristics.

	Patients			Controls
	Whole Cohort N = 52	One Booster (V3) N = 40	Two Boosters (V4) N = 12	One Booster (V3) N = 12
**Period of analysis**	From 18 January 2022 to 3 March 2022			26 January 2022 to 4 February 2022
**Gender:** Male/female	33/19	26/14	7/5	3/9
**Median age:** years (range)	54 (20–74)	54 (20–74)	55 (25–69)	54 (39–63)
**Underlying disease:**				NA
AML/MDS/MPS (myeloid)	20/10/6 (36)	11/7/6 (24)	9/3/0 (12)	
ALL/NHL/HL/MM (lymphoid)	8/4/3/1 (16)	8/4/3/1 (16)	0/0/0 (0)	
**Donor type:**				NA
Geno-identical/MUD/haplo/9/10 mis-MUD	15/22/13/2	12/18/8/2	3/4/5/0	
**Conditioning:**				NA
Myeloablative/reduced-intensity/sequential	8/41/3	8/30/2	0/11/1	
**GVHD prophylaxis:**				NA
CsA+ ATG (+-MMF or methotrexate)	28	26	2	
CsA + MMF + PTCY	13	7	6	
PTCY only	11	7	4	
**Previous GVHD:** No/Yes	20/32	15/25	5/7	NA
**Ongoing treatment:** No/Yes *	41/11	32/8	9/3	

Abbreviations: AML: acute myeloid leukemia; MDS: myelodysplastic syndrome; MPS: myeloproliferative syndrome; ALL: acute lymphoblastic leukemia; NHL: non-Hodgkin lymphoma; HL: Hodgkin lymphoma; MM: multiple myeloma; MUD: matched unrelated donor; GVHD: graft-versus-host disease; CsA: cyclosporine A; ATG: antithymoglobulin; MMF: mycophenolate mofetyl; PTCY: post-transplant cyclophosphamide; V1: first vaccine; V3: third vaccine = first booster; V4: fourth vaccine = second booster. *: immunosuppressive drugs for active GVHD: CsA (+ corticosteroid) *n* = 4 (2); corticosteroid alone *n* = 1; ruxolitinib (+ cortisteroid) *n* = 4 (1); or chemotherapy: ponatinib as relapse prophylaxis *n* = 1; vincristine+ corticosteropid for relapse *n* = 1.

**Table 2:** Anti-SARS-CoV-2 vaccinations and anti-spike responses.

	Patients			Controls
	Whole Cohort N = 52	One Booster (V3) N = 40	Two Boosters (V4) N = 12	One Booster (V3) N = 12
**Type of vaccine**				6/6
BNT162b1/ mRNA-1273	52/0	40/0	12/0	
**Median time from transplant to V1**				NA
days (range)	389 (86–4939)	692 (91–4939)	126.5 (86–1000) *p* = 0.001	
**Median time from transplant to**:				NA
V3 days (range)	487 (143–5160)	889 (167–5160)	267 (143–1090) *p* = 0.001	
V4: days (range)			459 (239–1306)	
**Median time from transplant to humoral and T-cell responses analyses:** days (range)	755 (189–5293)	1045 (189–5293)	517 (265–1376) *p* = 0.008	NA
<1 year	9	5	4	
1–2 years	18	11	7	
>2 years	25	24	1	
**Median time from V1 to humoral and T-cell responses analyses**: days (range)	330 (84–404)	324 (84–398)	367 (175–404) *p* = 0.44	381 (358–391) *p* = 0.004
**Median time from V3 to humoral and T-cell responses analyses**: days (range)	229 (12–298)	224 (12–298)	255 (113–296) *p* = 0.19	55 (18–74)
**Median time from last vaccine to humoral and T-cell responses analyses**: days (range)	125 (12–298)	224 (12–298)	60 (16–117) *p* = 0.0006	55 (18–74) *p* = 0.002
**Humoral response after V3/V4 N (%):**				
Antibodies < 0.8 BAU/mL	5 (9.5%)	4 (10%)	1 (8%)	0
Antibodies 0.8–250 BAU/mL	5 (9.5%)	3 (7.5%)	2 (17%)	0
Antibodies > 250 BAU/mL	42 (81%)	33 (82.5%)	9 (75%) *p* = 0.63	12 (100%) *p* = 0.25
Antibodies > 2500 BAU/mL	27 (52%)	20 (50%)	7 (58%) *p* = 0.85	12 (100%) *p* = 0.005
**T-cell response after V3/V4: N (%)**	44 (85%)	34 (85%)	10 (83%) *p* = 1	12 (100%) *p* = 0.33
Median % of anti-spike T-cells (range) **	0.034 (0–1.143)	0.028 (0–0.771)	0.063 (0–1.143) *p* = 0.21	0.127 (0.006–0.235) *p* = 0.08

** expressed as SFU/100 CD3+ T cells. *p* in “two boosters” column: comparison between patients receiving one or two boosters. *p* in “controls” column: comparison between whole cohort and controls.

## Data Availability

Full data are available upon request.

## Supplementary Materials

The following supporting information can be downloaded at: https://www.mdpi.com/article/10.3390/cells11193010/s1, Table S1: Factors predicting T-cell response: Univariate analysis. Table S2: Vaccinated patient characteristics according to humoral responses; Figure S1: CD4+ and CD8+ T-cell polyfunctional cytokine profile.
