# Supplementary material for: Strong SARS-CoV-2 T-Cell Responses after One or Two COVID-19 Vaccine Boosters in Allogeneic Hematopoietic Stem Cell Recipients
Source: Cells. 2022 Sep 27;11(19):3010. doi: 10.3390/cells11193010 (PMC9563037; doi:10.3390/cells11193010)
Supplement: Supplementary file 1 [file cells-11-03010-s001.zip › cells-1906823-SM.pdf]

Article

Strong SARS-CoV-2 T-cell Responses after One or Two COVID-19 Vaccine Boosters in Allogeneic Hematopoietic Stem Cells Recipients

Béatrice Clémenceau,[1] Amandine Le Bourgeois,[2] Thierry Guillaume,[1,2] Marianne Coste-Burel,[3] Pierre Peterlin,[2] Alice Garnier,[2] Maxime Jullien,[2] Jocelyn Ollier,[1] Audrey Grain,[1] Marie C Béné,[1,4] Patrice Chevallier,[1,2]

Supplemental files

Table S1: **Factors predicting T-cell response: Univariate analysis**

Factors	Non responders n=8	Responders n=44	P value
Number of vaccines received: 3/4	6/2	34/10	1
Anti-spike antibody rate: $\leq$250/>250 BAU/mL	2/6	8/36	1
Gender: male/female	8/0	25/19	0.053
Median age: years (range)	57 (49-71)	53 (20-74)	0.84
Disease: lymphoid/myeloid	3/5	13/31	0.97
Donor type: geno/MUD/haplo/mism	1/5/2/0	14/17/11/2	0.54
Conditioning: MAC/RIC/Sequential	2/6/0	6/35/3	0.56
GVHD prophylaxis: ATG/Csa+MMF+PTCY/PTCY alone	5/2/1	23/11/10	0.79
Previous GVHD: no/yes	3/5	17/27	1
Ongoing treatment: no/yes	7/1	34/10	0.85
Median delay V3/V4-analyses: days (range)	94 (12-295)	136 (16-298)	0.46
Median delay graft-analyses: days (range)	703 (356-4047)	755 (189-5293)	0.70
Lymphocytes: 10^9/L (range)	2.66 (0.75-3.96)	1.37 (0.12-7.14)	0.053
Gamma globulins: g/L (range)	7.7 (3.4-17.3)	6.75 (1.1-13.9)	0.53

Table S2: **Vaccinated patient characteristics according to humoral responses**

	Allogeneic hematopoietic stem-cell recipients (N=52, 3 doses n=40; 4 doses n=12) Antibody response after V3/V4			
	**Whole Cohort**	**No (<0.8 BAU/mL) n=5**	**Low (0.8-250 BAU/mL) n=5**	**High (>250 BAU/L) n=42**
T-cell response after V3/V4 . Patients (%)	44 (85%)	3 (60%)	5 (100%)	36 (86%)
Median % (range) of anti-spike T cells	0.037 (0-1.143)	0.039 (0-0.641)	0.056 (0.002-0.771)	0.034 (0-1.143)
Median time from transplant to V1: days (range)	389 (86-4939)	207 (102-327)	228 (127-1402)	617 (86-4939)
Median time from transplant to V3/V4: days (range)	616 (167-5160)	395 (207-482)	287 (216-1551)	758 (167-5160)
Median time from transplant to humoral and T cells responses analyses: days (range)	755 (189-5293)	407 (401-649)	400 (238-1663)	988 (189-5293)
Median time from V1 to humoral and T cells responses analyses: days (range)	330 (84-404)	250 (161-322)	175 (98-397)	347 (84-404)
Median time from V3/V4 to humoral and T cells responses analyses: days (range)	125 (12-298)	50 (12-196)	74 (22-113)	217 (16-298)
**Gender:** Male/Female	33/19	3/2	2/3	28/14
**Median age**: years (range)	54 (20-74)	57 (32-71)	52 (34-59)	55 (20-74)
**Underlying disease:**				
AML/MDS/MPS (myeloid)	20/10/6 (36)	3/1/0 (4)	2/0/0 (2)	15/9/6 (30)
ALL/NHL/HL/MM (lymphoid)	8/4/3/1 (16)	0/0/1/0 (1)	2/1/0/0 (3)	6/3/2/1 (12)
**Donor type:**				
Geno-identical/MUD/Haplo/9/10 mis-MUD	15/22/13/2	0/4/1/0	1/3/1/0	14/15/11/2
**Conditioning:**				
Myeloablative/Reduced-intensity/Sequential	8/41/3	0/4/1	2/2/1	6/35/1
**GVHD prophylaxis:**				
CsA+ ATG (+-MMF or methotrexate)	28	4	3	21
CsA+MMF+PTCY	13	1	2	10
PTCY only	11	0	0	11
**Previous GVHD:** No/Yes	20/32	1/4	0/5	19/23
**Ongoing treatment:** No/Yes*	41/11	4/1	2/3	35/7

Abbreviations: V1: first vaccine; V3: third vaccine; V4: fourth vaccine; AML: acute myeloid leukemia; MDS: myelodysplastic syndrome; MPS: myeloproliferative syndrome; ALL: acute lymphoblastic leukemia; NHL: non-Hodgkin lymphoma; HL: Hodgkin lymphoma; MM: multiple myeloma; MUD: matched unrelated donor; GVHD : graft-versus-host disease; CsA: cyclosporine; MMF: mycophenolate mofetyl; PTCY: post-transplant Cyclophosphamide; NA: not applicable.

*: immunosuppressive drugs for active GVHD: CsA (+ corticosteroids)n=4 (2); corticosteroids alone n=1; ruxolitinib (+ cortisteroids) n=4 (1); or chemotherapy: ponatinib as relapse prophylaxis n=1; vincristine+ corticosteroids for relapse n=1.

Supplemental Figure S1

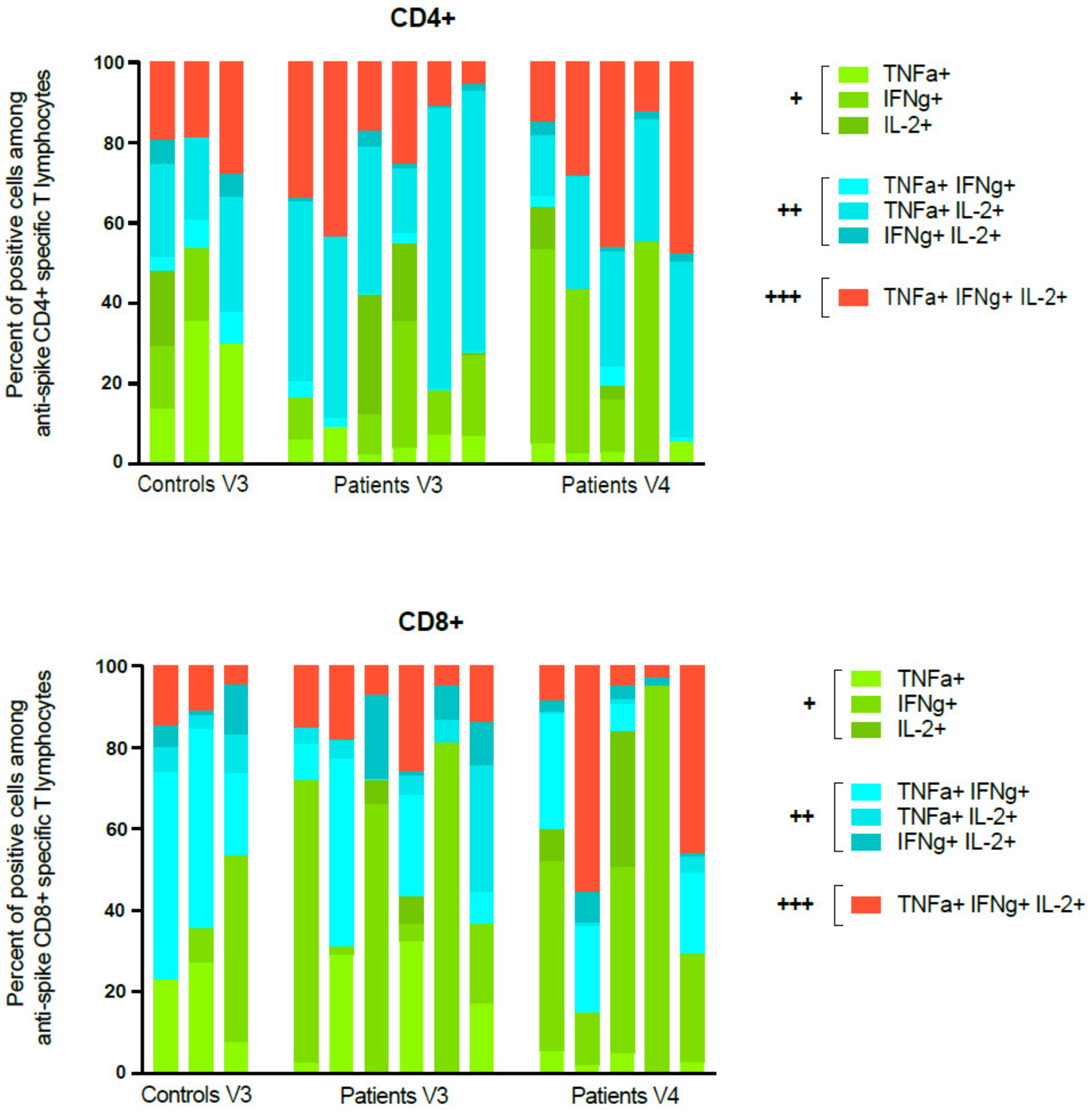

Figure S1: (A) CD4+ and (B) CD8+ T-cell poly-functionality analysis. Bars represent the proportion of T-cells displaying each particular cytokine profile (7 possible combinations of positive responses). Colors depict the distribution of mono-(green), bi-(blue) and tri-(red) cytokine-producing T cells.
